# Rationale‐based selection of optimal operating strategies and gene dosage impact on recombinant protein production in *Komagataella phaffii* (*Pichia pastoris*)

**DOI:** 10.1111/1751-7915.13498

**Published:** 2019-10-28

**Authors:** Miguel Angel Nieto‐Taype, Javier Garrigós‐Martínez, Marc Sánchez‐Farrando, Francisco Valero, Xavier Garcia‐Ortega, José Luis Montesinos‐Seguí

**Affiliations:** ^1^ Department of Chemical, Biological and Environmental Engineering School of Engineering Universitat Autònoma de Barcelona 08193 Bellaterra Spain; ^2^ Present address: Purac Bioquímica S.A. (Corbion Montmeló) Montmeló Spain

## Abstract

Its features as a microbial and eukaryotic organism have turned *Komagataella phaffii* (*Pichia pastoris*) into an emerging cell factory for recombinant protein production (RPP). As a key step of the bioprocess development, this work aimed to demonstrate the importance of tailor designing the cultivation strategy according to the production kinetics of the cell factory. For this purpose, *K. phaffii* clones constitutively expressing (*P*_GAP_) *Candida rugosa* lipase 1 (Crl1) with different gene dosage were used as models in continuous and fed‐batch cultures. Production parameters were much greater with a multicopy clone (MCC) than with the single‐copy clone (SCC). Regarding production kinetics, the specific product generation rate *(q*_P_ *)* increased linearly with increasing specific growth rate (*µ*) in SCC; by contrast, *q*_P_ exhibited saturation in MCC. A transcriptional analysis in chemostat cultures suggested the presence of eventual post‐transcriptional bottlenecks in MCC. After the strain characterization, in order to fulfil overall development of the bioprocess, the performance of both clones was also evaluated in fed‐batch mode. Strikingly, different optimal strategies were determined for both models due to the different production kinetic patterns observed as a trade‐off for product titre, yields and productivity. The combined effect of gene dosage and adequate *µ* enables rational process development with a view to optimize *K. phaffii* RPP bioprocesses.

## Graphical abstract

The combined effect of strain and bioprocess engineering should be considered during a rational process development towards bioprocess optimization. The synergy between gene dosage and specific growth rate have been evaluated for the *Komagataella phaffii* (*Pichia pastoris*) recombinant protein production (RPP) of an industrial lipase in chemostat and fed‐batch cultures. Different optimal strategies were designed from a trade‐off for product titer, yields and productivity for two clone models with different gene dosage and production kinetics, from which further transcription analysis supported potential bottlenecks after transcription.

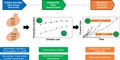

## Introduction

*Komagataella phaffii,* formerly known as *Pichia pastoris*, is a widely used yeast for recombinant protein production (RPP), for both biopharmaceuticals and an increasing number of industrial enzymes of interest (Puxbaum *et al.*, 2015; Burgard *et al.*, 2017). This host has major advantages such as a wide range of genetic modification tools including genome editing toolkits are available (e.g. the CRISPR/Cas9 system); its ability to grow to a high cell density in defined media, to perform eukaryotic post‐translational modifications and to release target products extracellularly. These features in combination make *K. phaffii* a promising cell factory for industrial biotechnology (Potvin *et al.*, 2012; Vogl and Glieder, 2013; Weninger *et al.*, 2018).

The increasing demand for recombinant proteins has generated a multibillion‐dollar market over the last few decades (Highsmith, 2015; Dewan, 2017). Therefore, important efforts are being dedicated to increase bioprocess efficiency and profitability. Two widely reviewed complementary approaches are currently being developed to reach these goals, namely strain engineering (Zahrl *et al.*, 2017; Juturu and Wu, 2018; Vogl *et al.*, 2018a) and bioprocess optimization (Theron *et al.*, 2018; Yang and Zhang, 2018).

Using efficient promoters is essential to ensure efficient recombinant protein expression in this context. The methanol‐inducible alcohol oxidase promoter (P_AOX1_) has been widely used in *K. phaffii* bioprocesses by virtue of allowing a strong and tight regulation for the recombinant expression in the presence of methanol (Barrigón *et al.*, 2013; Ponte *et al.*, 2016; Vogl *et al.*, 2016). However, P_AOX1_‐driven expression bioprocesses are subject to constraints derived from the use of methanol as inducer. Thus, using methanol at the industrial scale requires adopting safety measures that raise production costs and is subject to operational problems arising from heavy high heat production and oxygen demand (Prielhofer *et al.*, 2013), cell metabolic burdens (Hartner and Glieder, 2006), culture cell lysis and potential subsequent proteolysis of the target product (Mattanovich *et al.*, 2009).

Alternative promoters avoiding the use of methanol have recently been explored (Liang *et al.*, 2013; Prielhofer *et al.*, 2013; Shen *et al.*, 2016; Vogl *et al.*, 2018b; Robert *et al.*, 2019). The glyceraldehyde‐3‐phosphate dehydrogenase *GAP* promoter (P_GAP_), which is involved in a key step of the glycolysis pathway, was the first to emerge as a benchmark for efficient protein expression on various carbon sources. Thus, by avoiding all methanol‐related drawbacks, P_GAP‐_based bioprocesses present relevant advantages for large‐scale production (Zhang *et al.*, 2009; Ahmad *et al.*, 2014; Çalık *et al.*, 2015).

Some authors have found copy number integration of the expression cassette in the genome, also called gene dosage, to play a central role in specific productivity (Schwarzhans *et al.*, 2016a; Vogl *et al.*, 2018a). Using large numbers of gene copies results in increased productivity in some cases (Nordén *et al.*, 2011; Prielhofer *et al.*, 2013; Zhu *et al.*, 2014) but has the opposite effect in others (Zhu *et al.*, 2009; Liu *et al.*, 2014; Cámara *et al.*, 2016).In fact, as claimed, integrating several expression cassettes in the genome may have adverse effects owing to the physiological limitations in the transcriptional capacity of gene *AOX1*, which is governed by its transcriptional factors (Cámara *et al.*, 2017).

Production kinetics, the relationship between specific production rate (*q*_P_) and specific growth rate (*μ*), is considered a key factor to be considered in the bioprocess development. It reflects the equilibrium between the various steps until the product is secreted, as a balance of the different processes involved during the protein synthesis, folding and secretion. This relationship is crucial to bioprocess development and optimization (Potvin *et al.*, 2012; Looser *et al.*, 2015; Çalik et al., 2015). Thus, Garcia‐Ortega *et al. *(2016) and Maurer *et al. *(2006) characterized P_GAP_‐based strains producing an antibody fragment and obtained robust results with them in chemostat systems; so, they found *q*_P_ to increase eight times with increasing *μ.* Rebnegger *et al. *(2014) examined the response of this expression system producing human serum albumin (HSA) at different specific growth rates at transcriptomic level and observed marked upregulation of genes involved in translation. However, genes of the glycolytic pathways such as *TDH3*, which is the endogenous gene regulated by P_GAP_, were unregulated or weakly regulated, therefore suggesting that effect in the translational machinery played a major role in by causing *q*_P_ to increase with increasing *μ.* The results obtained with some fed‐batch cultures are also consistent with synergism in these two variables (Zhao *et al.*, 2008; Garcia‐Ortega *et al.*, 2013). Therefore, because gene dosage is expected to affect production rates, one should assume that it may considerably influence production kinetics in assessing its effects.

*Candida rugosa* lipase (Crl) is one of the most promising lipase enzymes for biocatalytic applications (Ken Ugo *et al.*, 2017). At least seven genes of *C. rugosa* lipases (*CRL1‐CRL7*) have been identified; also, all except *CRL6* and *CRL7* have been identified and sequenced (Ferrer *et al.*, 2001). Crl1, which accounts for about 80% of all lipase present in commercial powders, is the most widely studied (Sánchez *et al.*, 1999) Because of the difficulty involved in isolating the pure isoenzyme from the native microorganism, it has been alternatively obtained from *K. phaffii* cell factory (Valero, 2018).

For the present work, the isoenzyme *Candida rugosa* lipase 1 (Crl1) was selected as model protein to elucidate the differences in the rational design of optimal bioprocess strategies for two clones of *K. phaffii* with different gene dosage, as example of clone variability in terms of protein production. For this purpose, an accurate characterization of physiological parameters and the production kinetics was firstly performed on chemostat cultures. The obtained results allowed the selection of the optimal bioprocess strategy to maximize the RPP, in order to be applied further in fed‐batch cultivations, which is currently considered as the most used operational mode for industrial RPP (García‐Ortega *et al.*, 2019). As a major outcome, this contribution discusses the influence of gene dosage linked to production kinetics on the determination of the optimal operating strategies with a view to maximizing bioprocess production rates and yields.

## Results and discussion

### Strain construction and gene dosage

In order to get two clones with contrasting production performances, several recombinant clones with different gene dosage of *CRL1* were obtained by transforming different amounts of plasmid in which the *CRL1* expression cassette is placed under P_GAP_ regulation. According to the transformation method used, these cassettes are expected to be integrated by homologous recombination into the native P_GAP_ locus. However, constructing producer strains from *K. phaffii* may result in non‐homologous end‐joining recombination and/or multiple insertion of the gene expression cassette, which usually expands the spectrum of clonal variability (Schwarzhans *et al.*, 2016b; Jiao *et al.*, 2018; Vogl *et al.*, 2018a).

Later, transformed clones were screened in order to identify the best producer clone, which was a clone that integrated five copies of the expression cassette. Thus, it was selected for further studies in which it was compared with a clone with a single copy of gene of interest*.* The determination of gene dosage was performed by ddPCR. This method allows to determine the exact number of expression cassettes that were integrated in multicopy clone (MCC), five copies, and to confirm the presence of only one copy of *CRL1* gene in the single‐copy clone (SCC).

### Strain characterization in chemostat cultures

#### Cell growth

**Figure 1:**
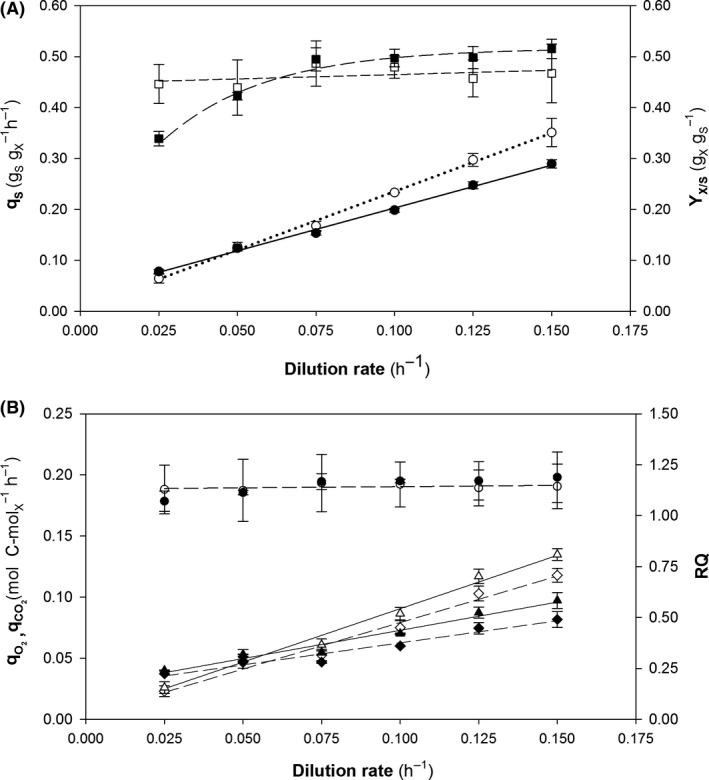
Main physiological parameters for continuous cultures of multicopy (black symbols) and single‐copy clones (white symbols). A. (●, ○) specific glucose uptake rate (*q*_S_); (■, □) overall biomass‐to‐substrate yield *Y*_X/S_. B. (♦, ◊) specific oxygen uptake rate ($q_{O_{2}}$); (▲, Δ) specific carbon dioxide production rate ($q_{{\text{CO}}_{2}}$); and (●,○) respiratory quotient (RQ). Error bars represent SE of the mean values.

The impact of gene dosage on clone production kinetics was assessed with two sets of chemostat cultures grown at different dilution rates (*D*). The specific growth rates spanned the range of 0.025–0.15 h^−1^. The carbon and electron balances were verified and closure found to exceed 95% prior to reconciliation. Fig. 1A and B show the variation of the main physiological variables at different *D* in both strains.

As can be seen in Fig. 1A, the specific substrate uptake rate (*q*_S_) increased linearly with increasing *μ*. Interestingly, there were no significant differences in *q*_S_ between clones, which suggests that this rate was unaffected by gene dosage. On the other hand, the overall biomass substrate yield (*Y*_X/S_) remained fairly constant with a value of about 0.5 g_X_ g_S_^–1^, which is consistent with most reported values (Garcia‐Ortega *et al.*, 2013; Çalik et al., 2015; Adelantado *et al.*, 2017). However, MCC exhibited a slight decrease in *Y*_X/S_ at the lowest *μ* values (0.05 and 0.025 h^‐1^) possibly due to requirement of more energy for maintenance (*m*_s_) than the single‐copy strain (0.029 vs. 0.0023 g_S_ g_X_^–1^ h^–1^). This factor strongly influenced *Y*_X/S_, which is consistent with some previous studies where biomass production decreased with decreasing *μ* (Rebnegger *et al.*, 2016).

The lower values observed in maintenance coefficient could be expected since *K. phaffii* has been described as a robust system in terms of present lower maintenance requirements over other alternative platforms such as *E. coli* (Zhu *et al.*, 2019). However, in this work, a relevant difference on *m*_S _has been described between SCC and MCC, specifically the difference is about one order of magnitude (0.0023 and 0.029 respectively). This notable change could be related to the RPP. It exerts a strong effect on metabolic fluxes that often leads to an increase in the maintenance requirements (Carnicer *et al.*, 2012; Moser *et al.*, 2017).

Accordingly, when comparing MCC respect to SCC in relative terms, the cell maintenance requirements consume a higher proportion of the overall energy resources obtained from carbon source uptake.

In Fig. 1B is shown how the specific O_2_ uptake ($q_{O_{2}}$) and CO_2_ production ($q_{{\text{CO}}_{2}}$) rates increased linearly with *μ*. Slight differences between both strains were observed at high *μ*. However, the proportion between these two specific rates are constant, and consequently, the respiratory quotient (*RQ*) was always about 1.15.

Similarly to *q*_S_, $q_{O_{2}}$ and $q_{{\text{CO}}_{2}}$ showed also to be strongly coupled with *μ*, therefore, fit into a linear equation pattern (*Herbert, Pirt, Luedeking‐Piret*), which describes how is distributed a determined bioprocess parameter (specific rate) for cell growth and maintenance.

Regarding the maintenance coefficient, which is represented by the intercept, for both specific rates of each clone, a rather similar value was obtained. Like *q*_S _(Fig. 1A), MCC maintenance coefficient was slightly higher, suggesting that this difference is produced due to the gene dosage effect. Thus, considering that five functional *CRL1* copies are integrated on MCC, it exerts a relevant demand of resources in comparison with SCC.

On the other hand, if it is compared $q_{O_{2}}$ and $q_{{\text{CO}}_{2}}$ trend for SCC and MCC*,* both display similar values across the *μ* until 0.10 h^‐1^. Nevertheless, at higher *μ*’s, a relatively slight decrease is detected for MCC in front of SCC. It has to be considered that RPP consumes energetic resources that drain precursors from the central carbon metabolism to sustain the productivity, which probably results in a readjustment of metabolism, being more inefficient (Peña *et al.*, 2018). For the MCC, it can be hypothesized that at low *μ*, Crl1 synthesis does not produce any significant metabolic readjustment; therefore, not significant changes are observed on gas‐related specific rates. However, at higher *μ* this readjustment is shown as a reduction in the oxygen consumption and carbon dioxide production rates.

#### Target protein production

**Figure 2:**
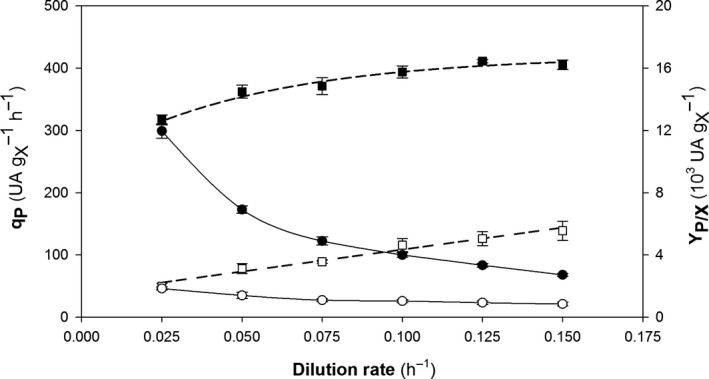
Production parameters for chemostat cultivation at different dilution rates of the multicopy (black symbols) and single‐copy clones (white symbols). (■, □) Specific product generation rate (*q*_P_); (●, ○) overall product‐to‐biomass yield coefficient (*Y*_P/X_). Error bars represent SE of the mean values.

The specific product generation rate (*q*_P_) and the overall product‐to‐biomass yield (*Y*_P/X_) were evaluated as main key production parameters. Both are shown in Fig. 2.

Most of the studies involving P_GAP_ have shown *q*_P_ to increase linearly with increasing *µ* (Khasa *et al.*, 2007; Rebnegger *et al.*, 2014; Garcia‐Ortega *et al.*, 2016). In the present work, similar behaviour was observed for the SCC. In the previous studies, heterologous protein expression was strongly coupled with cell growth. With the methanol‐inducible promoter, *P*_AOX1_, *q*_P_ may not increase with increase in *µ* and substrate inhibition may arise as a result (Cos *et al.*, 2006; Ahmad *et al.*, 2014; Schwarzhans *et al.*, 2017; Ponte *et al.*, 2018).

As expected, *q*_P_ was greater with MCC than with SCC (roughly 3–6 times). Some studies have shown protein production to be correlated with gene dosage and hence suggest that the number of gene copies influences *q*_P_ up an optimum number above which the synergistic effect usually is lost (Schwarzhans *et al.*, 2016a; Betancur *et al.*, 2017; Dagar and Khasa, 2018; Vogl *et al.*, 2018a).

Unlike SCC, the variation of *q*_P_ for MCC with *µ* was not linear. In fact, a saturation effect was observed at *µ *> 0.10 h^–1^, from which the *q*_P_ reaches a rather constant value at about 390 UA·g_X_^−1^ h^−1^. As a result, the proportional difference in *q*_P_ between the two clones decreased with increasing *µ* (from roughly six times in the low *µ* range to only about three times in the high *µ* range).

During the chemostat cultures, the stability of the strains has been demonstrated by confirming the gene copy number of the expression cassette genome integration by ddPCR. Other works also have evaluated strain stability for *K. phaffii* strains in long‐run chemostats describing a high genetic stability of the recombinant strains (Cankorur‐Cetinkaya *et al.*, 2018).

Looser *et al.* (2015) described that the strains expressing heterologous proteins under P_GAP_ normally show a linear pattern of production kinetics. Therefore, the optimum *µ* value for production purposes must be close to *μ*_max_. However, in this work *q*_P_ for MCC results exhibited saturation at specific growth rates lower than *μ*_max_, so identifying the optimum *µ* value should not be straightforward, which was also suggested by other authors (Maurer *et al.*, 2006; Buchetics *et al.*, 2011).

As shown in Fig. 2, the variation of *Y*_P/X_ with *µ* differed between SC and MC clones. While the variation pattern for *Y*_P/X_ in SCC was quite constant irrespective of *D*, *Y*_P/X_ in MCC exhibited a substantial decrease in the higher *D* range. Thus, the highest values of *Y*_P/X_ were obtained for low specific growth rates (0.025 and 0.05 h^–1^).

Protein production kinetics reflects the equilibrium between the various steps involved in the synthesis, folding and secretion of proteins, which are influenced by a variety of physiological factors. Based on the results for MCC, one may hypothesize that some of the processes involved in protein production are subject to a bottleneck in the high *µ* range that leads to the saturation effect observed in the production kinetic profile. This result is consistent with previous reports where the expression system seemingly saturated during transcription, protein synthesis, post‐translational modifications or even secretion (Puxbaum *et al.*, 2015; Cámara *et al.*, 2017). Further research is therefore required to improve existing knowledge about the events. In this work, we focused the efforts on the transcriptional analysis of key genes.

### Transcriptional analysis

As suggested in the previous section, further research into subjects such as transcriptional analysis was deemed necessary to throw further light onto the differences in *q*_P_ variation patterns between SC and MC clones in chemostat cultures. For this purpose, relative transcript levels in relevant target genes were determined under all the culture conditions studied in chemostat cultures, using qPCR as described in the experimental procedures section.

**Figure 3:**
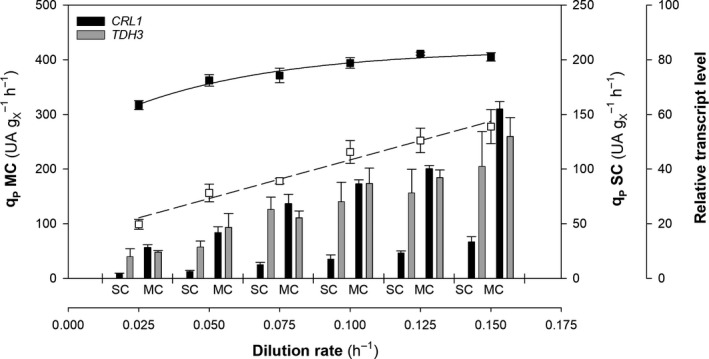
Specific product generation rate, *q*_P_, of the single‐copy (□) and multicopy clones (■); and relative transcript level of the clones for *CRL1* (black bars) and *TDH3* (grey bars) at each dilution rate. Error bars represent SE of the mean values.

Figure 3 compares the correlation of *q*_P_ with the relative transcript levels of the genes *CRL1* and *TDH3*. Initially, *TDH3* transcription increased linearly with increasing *D* in both clones regardless the *CRL1* gene dosage. This result can be ascribed to the *TDH3* product corresponding to a critical node in the glucose uptake pathway, which is closely associated with biomass growth (Nocon *et al.*, 2014; Çalik et al., 2015). Consequently, with high specific biomass growth rates, high fluxes through the glycolytic pathway can only be maintained by increasing production in *TDH3*. These results would be in contrast with those microarrays results presented by Rebnegger *et al. *(2014) in which the *TDH3* transcription appeared to be unregulated with the dilution rate. Remarkably, it is considered that qPCR is thought to provide increased quantitative resolution in transcriptional analyses of genes that present slight differences among samples (Morey *et al.*, 2006). Finally, on constancy of dilution rate (*D*), *TDH3* relative transcript levels were essentially identical for the two strains, which suggest that the levels were influenced by *D* but not by *CRL1* gene dosage.

Consistent with the results of the enzymatic activity analysis, *CRL1* gene expression was initially greater in MC than in SC clones. In SCC, *CRL1* relative transcript levels increased linearly with increasing *D*, which suggests coupling of *µ*, *CRL1* relative transcript levels and *q*_P_. These results indicate that P_GAP_ regulates genes *TDH3* and *CRL1* similarly and hence that the effects are strongly related with cell growth. This was not the case with MCC, however. Thus, although *CRL1* gene expression was seemingly not affected by *D*, *q*_P_ clearly saturated at high *D* levels. This may have resulted from *CRL1* expression coupling with biomass growth and the total amount of product secreted being limited. Therefore, there might be a bottleneck after transcription precluding conversion of all *CRL1* transcripts into proper folded and secreted Crl1.

**Figure 4:**
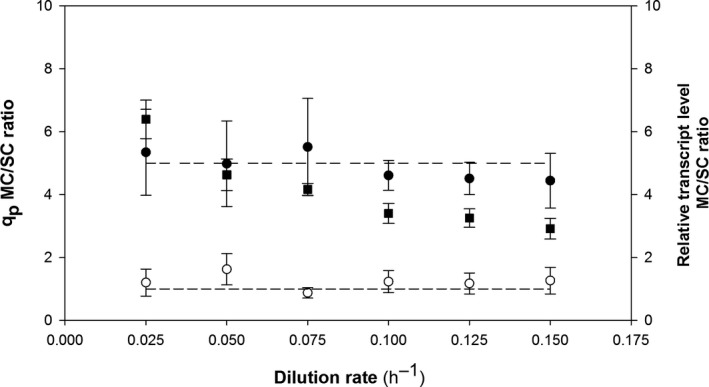
Product and relative transcript‐level ratios between multicopy and single‐copy clones at different dilution rates in chemostat cultures. (■) Ratio of specific product generation rates (*q*_P_); (●) ratio of relative transcript level for *CRL1*; and (○) ratio of relative transcript level for *TDH3*. Error bars represent SE of the mean values.

In Fig. 4, in which the transcriptional regulation of *TDH3* and *CRL1* for different *µ* is compared by means of plotting the quotient of the relative transcript levels between the MCC and the SCC, can be observed the above‐mentioned trends. Interestingly, while no effect was observed for the *TDH3* quotient, the positive effect of *CRL1* gene dosage in terms of transcription is confirmed for all the culture conditions tested. According to these results, therefore, no limitation in the transcriptional machinery for *CRL1* can be stated. Nevertheless, and despite the *CRL1* quotient of transcript levels remains constant regardless the different cultures conditions at different *D*, there is a significant decrease in the *q*_P_ quotient when increasing *µ*. This would support the hypothesis that the relevant increases achieved in terms of transcript levels due to the higher gene dosage of the target gene cannot be therefore converted into functional protein of interest.

Strikingly, the gene dosage effect observed in this P_GAP_‐driven example of protein expression differs markedly from that reported by Cámara *et al. *(2017) for P_AOX1_‐driven *Rhizopus oryzae* lipase (Rol) recombinant expression. The increased number of cassettes in Rol attenuated transcription of methanol metabolism and decreased methanol consumption, cell growth and recombinant protein production as a result.

Because *K. phaffii* may be limited in terms of protein folding, glycosylation and secretion, reduced product yields and productivity could be expected. These limitations were widely discussed by Puxbaum *et al. *(2015) and are related to ER‐associated degradation (ERAD), ER‐Golgi trafficking in the secretory pathway and unfolded protein response (UPR). There have been some attempts at circumventing these constraints and enhancing production of various recombinant proteins. For instance, some authors have used increased gene dosages of target genes involved in product folding and secretion such as *HAC1*, *PDI1* and/or *KAR2* (Bankefa *et al.*, 2018; Guan *et al.*, 2016; Liu *et al.*, 2014; Yang *et al.*, 2016). Other authors (Barrero et.al., 2018) have engineered the secretion signal in order to improve translocation into the endoplasmic reticulum (ER).

No limitation in *TDH3* or *CRL1* gene transcription was observed here. With MCC, however, *CRL1* relative transcript levels increased linearly with increasing *D,* but the transcripts could not be converted into functional secreted Crl1 owing to potential post‐transcriptional constraints. In order to shed further light on the presumed bottlenecks, the relative expression of other target genes including *PGK*, *KAR2* and *HAC1* was examined. No limitation in chemostat cultures was observed at any *D* value, so we can conclude that the specific growth rate had no regulatory effect on the expression of these genes. Overall, this result reveals that, although no transcriptional limitation was identified – and hence *CRL1* gene transcription in MCC increased linearly with *D* – this trend was inconsistent with that in the amount of product formed and hence suggests the presence of post‐transcriptional constraints.

### Fed‐batch cultures

In addition to the chemostat cultures, the performance of SC and MC clones was also examined in fed‐batch cultures, which is the operational mode typically used for industrial RPP. All fed‐batch fermentations were conducted according to a carbon‐limited pre‐programmed exponential feeding profile in order to maintain a constant *µ* throughout the cultivation period. The ending criteria selected for these cultures were to reach a final biomass concentration about 100 g l^–1^ to compare the experiments with a similar biomass concentration and always below maximal working volume. Thus, the culture is stopped before the bioprocess may be limited due to some biological and physical restrictions, such as heat and mass transfer, and lack of homogeneity, keeping the pseudo‐stationary state in the system. *μ* spanned the range of 0.05–0.15 h^–1^ and included an additional condition of 0.025 h^–1^ for MCC.

**Figure 5:**
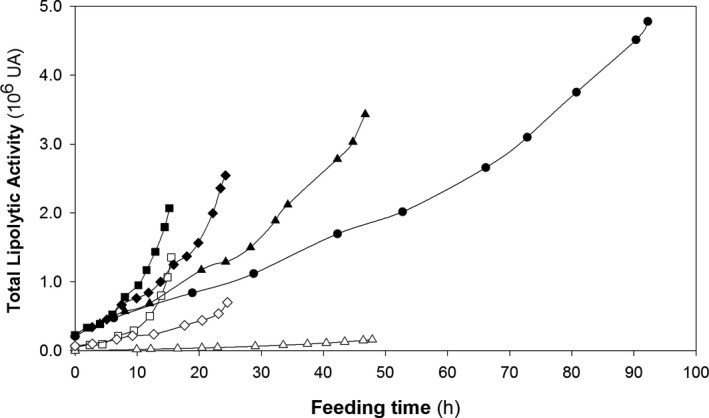
Variation of the total lipolytic activity in fed‐batch cultures at different specific growth rates (*µ*) with the multicopy (black symbols) and single‐copy clones (white symbols). Specific growth rates: (●) 0.025 h^–1^, (▲) 0.05 h^–1^, (♦) 0.10 h^–1^, (■) 0.15 h^–1^, (Δ) 0.05 h^–1^, (◊) 0.10 h^–1^ and (□) 0.15 h^–1^.

Figure 5 presents the time‐course of total lipolytic activity in the fed‐batch cultures with SC and MC clones. Although the figure only shows the results for the fed phase, it should be noted that lipolytic activity at the end of the batch phase was roughly 3.5 times higher with MC clone. Since biomass grew at the highest possible *µ* during the batch phase, these results seemingly confirm the effect of gene dosage on high *µ* cultures suggested in the previous section.

As detailed in Table 1, lipolytic activity peaked at 372 UA ml^–1^ at the highest *µ* value with SCC and at 1009 UA ml^–1^ at low *µ* (0.05 h^–1^) with MCC.

**Table 1:** Main production parameters obtained with the single‐copy and multicopy clones at different specific growth rates (*µ)* in fed‐batch cultures. For the comparing rows, cultivation at the lowest *µ* of the single‐copy clone is taken as reference.

	Single‐copy clone			Multicopy clone			
Nominal *µ* (h^−1^)	0.050	0.100	0.150	0.025	0.050	0.100	0.150
Experimental *µ* (h^−1^)	0.048	0.088	0.133	0.023	0.051	0.087	0.147
Product titre (UA ml^−1^)	180	195	372	1414	1009	827	600
Product titre increase (%)	−	+8%	+107%	+685%	+460%	+359%	+233%
*q*_P_ (UA g_X_^−1^ h^−1^)	77	149	292	374	550	697	777
*q*_P_ increase (%)	−	+92%	+277%	+383%	+611%	+800%	+903%
*Q*_P_ (10^3^ UA l^−1^ h^−1^)	3.3	6.3	22.9	14.6	20.1	31.1	34.9
*Q*_P_ increase (%)	−	+87%	+586%	+337%	+502%	+831%	+943%
*Y*_P/S_ (10^3^ UA g*_S_* ^−1^)	0.75	0.71	0.94	7.33	5.54	3.72	2.61
*Y*_P/S _increase (%)	−	−5%	+26%	+880%	+640%	+398%	+248%
*Y*_P/X_ (10^3^ UA g*_X_* ^−1^)	1.61	1.70	2.19	16.1	10.9	8.02	5.28
*Y*_P/X _increase (%)	‐	+6%	+36%	+902%	+577%	+399%	+229%

*µ,* specific growth rate; *q*_P_ *,* specific product generation rate; *Q*_P_, volumetric productivity; *Y*_P/S_, overall product‐to‐substrate yield; *Y*_P/X_, overall product‐to‐biomass yield.

Based on these results for MCC, an additional fermentation run at 0.025 h^–1^ was used to confirm that Crl1 production would peak at the lowest *μ* level. As hypothesized, lipolytic activity (1414 UA ml^–1^) was highest under those conditions and four times greater than the highest value for SCC. A systematic comparison based on the main production parameters obtained at the different operating conditions followed with the two producer clones is presented in Table 1.

**Figure 6:**
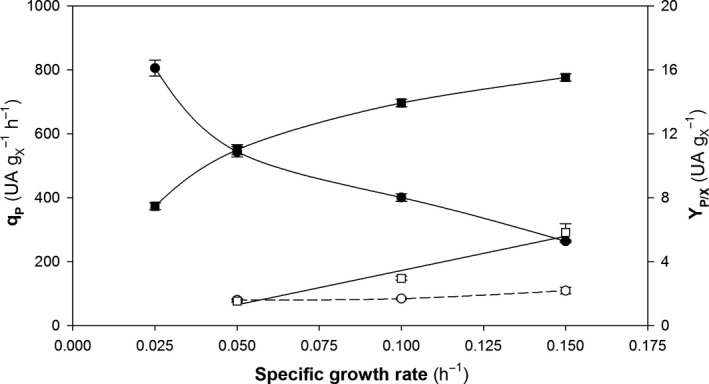
Production parameters for fed‐batch cultures at different specific growth rates of the multicopy (black symbols) and single‐copy clones (white symbols). (■,) Specific product generation rate (*q*_P_); (●, ○) overall product‐to‐biomass yield coefficient (*Y*_P/X_). Error bars show SE from regression analysis.

In Fig. 6, the mean values of *q*_P_ and *Y*_P/X_ for the fed‐batch cultures at different *µ* values are depicted. As expected for consistency with the chemostat results, *q*_P_ increased linearly with increasing *µ* in SCC but exhibited saturation in MCC. The *Y*_P/X_ variation pattern was similar to that for the chemostat cultures; thus, *Y*_P/X_ decreased exponentially with increasing *µ* in MCC, but remained fairly constant in SCC.

As in the chemostat cultures, *q*_P_ was higher with MCC than with SCC, and their ratio (3–6) was dependent on the *µ* applied. Although the overall patterns for the two operational modes were similar, *q*_P_ and *Y*_P/X_ were substantially higher for the fed‐batch cultures.

Fed‐batch cultivation usually ends when biomass production reaches a critical concentration limit imposed by biological and physical restrictions. As a result, growth‐associated biomass in fermentation processes is an unavoidable by‐product and requires controlling the biomass growth in order not to exceed the limiting concentration. Thus, the lower *μ* is, the longer a bioprocess can be extended to increase product titres until the termination criterion is fulfilled.

In Table 1 are gathered the main production parameters obtained in fed‐batch cultures for the single‐copy and multicopy clones. Product titre, yields and productivities are detailed as key performance indexes along the different specific growth rates applied. Some aspects can be assessed for discussion, mainly relating to the synergistic effect of *µ* and *q*_P_, and how these two specific rates affect the bioprocess efficiency.

Regarding the production kinetics, that is, how affects *μ* on *q*_P, _it was demonstrated that the kinetic pattern is markedly different for the MCC and SCC. The MCC exhibited a 30% *q*_P_ decrease when *μ* decreased from 0.15 to 0.05 h^–1^, by effect of its rather flat production kinetic curve at the highest *μ*, as shown in Fig. 6 and Table 1. In a similar comparison for the SCC, *q*_P _decreased by 70 % from 0.15 to 0.05 h^–1^. It is due to the more marked coupling of *q*_P_ to the cell growth.

As expected, productivities (*Q*_P_) varied on *µ* similarly to that observed for *q*_P_ in MCC and SCC. Consequently, the rational selection of *µ* to optimize the bioprocess performance would require a trade‐off.

In terms of parameters focused on production and yield, the SCC at 0.15 h^–1^ reached product titres 107 % higher compared with the obtained at 0.05 h^–1^. Accordingly, *Y*_P/S_ and *Y*_P/X_ increased a 26% and 36% respectively. Therefore, high *µ* should be clearly recommended for production kinetic patterns similarly to SCC.

On the contrary, for the MCC, product titre reached a 68 % increase when *μ* decreased from 0.15 to 0.05 h^–1^. Strikingly, titre was even better at 0.025 h^–1^, equivalent to a 140 % increase compared with 0.15 h^–1^, which lead to important increases in terms of *Y*_P/S_ and *Y*_P/X_ (180% and 204% respectively).

### Rational‐based bioprocess design

The results previously exposed support the idea that combined effect of *µ* and gene dosage on P_GAP_‐based *K. phaffii* clones expressing *CRL1* can be used to design optimal operating strategies.

In this way, the results obtained from chemostat cultures reveal that *q*_P_ increases linearly with increasing specific growth rate in SCC but exhibits a non‐linear pattern suggestive of saturation of the production kinetics in MCC. Interestingly, *q*_P_ is considerably higher with MCC than with SCC (3–6 times depending on the particular *µ* value). These results confirm that gene dosage impacts directly on the production kinetic profile, both in the levels of titre achieved as well as the profile pattern. From the transcriptional analysis performed, one can hypothesize the presence of a potential bottleneck after transcription.

The continuous cultures were used as tool to the design of optimized fed‐batch strategies, since this operational mode is the most widely operational mode used for industrial RPP. Therefore, a rational‐based bioprocess design can be carried out considering the effect of gene dosage and *μ* on the bioprocess efficiency. Although trends were similar as those observed in the chemostat cultures, there were some differences, especially with MCC. Saturation was more marked than in the chemostat tests, and a plateau was reached in the higher *µ* range*.*

When the data for the main production parameters in Table 1 are jointly considered, the different trends in production kinetics and yields can be useful to identify the best operational strategies by characterizing producer clones. Usually, when *µ* has a strong impact on specific production rates and yields (e.g. with SCC), a high *µ* level should be used to increase *q*_P_ and *Y*_P/X_. On the other hand, if the impact of *µ* is weak (e.g. with MCC), low‐to‐moderate *µ* values can help to extend the bioprocess time until the limit imposed by a critical amount of biomass is reached. In this case, these differences have been caused by the different gene dosage.

Summing up, it can be stated as main outcome of this contribution that the production kinetics of the cell factory and specific growth rate must be jointly considered to tailor operating strategies to production clones for maximizing expression in *K. phaffii*. This work presents how a comprehensively elucidation of the gene dosage effect on production kinetics towards an overall optimization of the RPP bioprocesses can be afforded. Hence, properly understanding these features and their correlation provides a more rational, robust knowledge of the behaviour of cell factories and allows bioprocesses to be engineered for greater efficiency.

## Experimental procedures

### Strains

Two recombinant clones of *K. phaffii* X‐33 from Invitrogen (Carlsbad, CA, USA) expressing *CRL1* regulated by the *GAP* promoter were obtained and used. A chimeric vector assembled with the restriction–ligation method was constructed by using commercial pGAPZαA plasmid from Invitrogen and a codon‐optimized synthetic *CRL1* coding sequence from GeneScript (Piscataway, NJ, USA). Different amounts of plasmid were transformed by electroporation in order to obtain clones with different numbers of integrated expression cassettes. However, only the clones containing 1 or 5 gene copies were used. Both clones can secrete Crl1 to the extracellular medium through the *Saccharomyces cerevisiae* α‐mating factor signal sequence.

### Gene copy number

Droplet digital PCR (ddPCR) was used to determine the number of cassette integrations in both clones, using a slightly modified version of the method of Cámara *et al. *(2016). Actin gene was used as housekeeping agent, but the primer sequence was that proposed by Landes et.al. (2016). The specific primers used are shown in Table S1.

### Transcriptional analysis

Transcriptional analyses were only done on chemostat cultures, where steady‐state conditions ensured homogeneity in cell population.

#### Total RNA extraction

Samples of 1 ml were withdrawn under different chemostat conditions and centrifuged at 4 ºC at maximum speed for 2 min. The resulting pellets were resuspended in 1 ml of TRIzol^TM^ (Waltham, MA, USA) and 200‐mg glass beads. Total RNA was extracted as per the manufacturer’s instructions. Then, RNA integrity and concentration were checked by agarose electrophoresis and Nanodrop analysis (Thermo Scientific^TM^, Waltham, MA, USA) respectively.

#### cDNA synthesis and transcriptional levels

cDNA was synthetized by using the iScript™ cDNA Synthesis Kit from Bio‐Rad (Hercules, CA, USA) according to the manufacturer’s instructions. A set of primers was designed to amplify cDNA for the target genes by qPCR. The set comprised *CRL1* (heterologous product); *TDH3*, which is the gene natively expressed under *P*_GAP_ control (glycolytic); and *PGK* gene, phosphoglycerate kinase (glycolytic). Additional genes involved in the unfolded protein response (UPR) and the secretory mechanisms such as *KAR2* and *HAC1* were also studied. Transcription was assessed by qPCR amplification. For maximum accuracy, mixes were made by the robot EpMotion® (Eppendorf, Germany). SYBR™ Select Master Mix was used as polymerase mix and a QuantStudio 12K Flex Real‐Timer from Thermo Scientific^TM^ for amplification cycles and data acquisition.

The qPCR programme was implemented as prescribed by the manufacturer but using a primer annealing temperature of 57.4°C. Relative transcript level was determined by using the *MTH1* glucose‐responsive transcriptional factors, which code for a negative regulator of the glucose‐sensing signal transduction pathway, as housekeeping agents. Rebnegger *et al. *(2014) previously found the specific growth rate to be uninfluential.

### Cultivation methods

Inoculum cultures for the bioreactor tests were prepared according to Garcia‐Ortega *et al. *(2013).

#### Chemostat cultivation

Chemostat cultures of the two strains were prepared in duplicate as described elsewhere (Garcia‐Ortega *et al.*, 2016). Different specific growth rates from 0.025 to 0.15 h^–1^ were evaluated. For every dilution rate, the continuous cultures were carried out for at least five residence times. In order to ensure that steady state was achieved, several samples were taken and analysed since three residence times during three consecutive residence times up to confirming the stability of the studied parameters.

#### Fed‐batch cultivation

Both strains were also cultivated in the fed‐batch mode, at different specific growth rates from 0.025 to 0.15 h^–1^. This strategy is based on a carbon‐limited feeding profile, keeping a constant *µ* during the culture reaching a pseudo‐stationary state. The process is described in detail elsewhere (Garcia‐Ortega *et al.*, 2013).

### Analytical methods

#### Determination of biomass as dry cell weight (DCW)

Biomass concentrations were measured in triplicate in terms of DCW as described elsewhere (Cos *et al.*, 2005). The relative standard deviation (RSD) was about 3%**.**

#### Quantification of the carbon source and by‐products

The concentrations of the different carbon sources used in the batch (glycerol) and fed‐batch tests (glucose), and of potential fermentation by‐products (arabitol or ethanol), were all determined by HPLC. The column and procedure used for this purpose are described elsewhere (Garcia‐Ortega *et al.*, 2017). RSD was always < 1%.

#### Off‐gas analyses

*BlueInOne Cell* gas analysers were used to monitor the cultures exhaust gas (BlueSens, Herten, Germany). CO_2_ and O_2_ mole fractions were measured on‐line with provision for off‐gas pressure and humidity. The data thus obtained were used to estimate the oxygen uptake rate (*OUR*), carbon dioxide evolution rate (*CER*), specific rates ($q_{O_{2}}$ and $q_{{\text{CO}}_{2}}$) and the respiratory quotient (*RQ*). RSD was < 5% in all cases.

#### Lipolytic activity assay

Crl1 activity was determined by using a modified version of an existing enzymatic assay based on *p*‐nitrophenyl butyrate (*p*NPB) (Chang *et al.*, 2006). The reaction buffer consisted of 1 mM *p*NPB, 50 mM phosphate buffer at pH 7 and 4 (v/v)% acetone. A volume of 980 µl of buffer was mixed with 20 µl of sample. The absorbance at 348 nm was measured on‐line at 30°C for 2 min on a *Specord 200 Plus* instrument from Analytic Jena (Jena, Germany). One unit of activity was defined as the amount of enzyme needed to release 1 mmol of *p*‐nitrophenol per minute under assay conditions. RSD was < 1%.

### Process parameters

#### Mass balance and stoichiometric equations

All equations derived from the mass balances used to calculate yields and rates in the chemostat (Garcia‐Ortega *et al.*, 2016) and fed‐batch cultures (Ponte *et al.*, 2016) were described elsewhere.

#### Data consistency and reconciliation

Measurement consistency was checked by using the standard test with carbon and electron balances as constraints. Both on‐line and off‐line measurements allowed five key specific rates in the black‐box process model to be calculated, namely biomass generation (*µ*), glucose uptake (*q_S_*), product generation (*q_P_*), oxygen uptake ($q_{O_{2}}$) and carbon dioxide production ($q_{{\text{CO}}_{2}}$). The methodology used is described in detail in a previous paper (Ponte *et al.*, 2016)**.**

## Conflict of interest

None declared.

## Supporting information


**Table S1**. Primer pairs used for gene dosage and transcript‐level determination by means of ddPCR and qPCR respectively.
