# Supplementary material for: Rationale‐based selection of optimal operating strategies and gene dosage impact on recombinant protein production in Komagataella phaffii (Pichia pastoris)
Source: Microb Biotechnol. 2019 Oct 28;13(2):315–27. doi: 10.1111/1751-7915.13498 (PMC7017824; doi:10.1111/1751-7915.13498)
Supplement: Supplementary file 1 — Table S1. Primer pairs used for gene dosage and transcript‐level determination by means of ddPCR and qPCR respectively. [file MBT2-13-315-s001.docx]

**Table S1.** Primer pairs used for gene dosage and transcript level determination by means of ddPCR and qPCR, respectively.

| **#** | **Name** | **Product** | **Function in analysis** | **Sequence** |
| --- | --- | --- | --- | --- |
| 1 | ACT_FW | ACT | ddPCR gene dosage housekeeping | CCTGAGGCTTTGTTCCACCCATCT |
| 2 | ACT_RE |  |  | GGAACATAGTAGTACCACCGGACATAACGA |
| 5 | MTH1_FW | MTH1 | qPCR transcription housekeeping | GGAACCTGGTCAACTGGGAACT |
| 6 | MTH1_RE |  |  | GTGGGAGGAGAACGATGTGGAA |
| 3 | CRL1_FW | CRL1 | Gene dosage and transcription | CCTGAGGGTACTTACGAAG |
| 4 | CRL1_RE |  |  | CCAGGTGGTCTAACAACG |
| 5 | KAR2_FW | KAR2 | Transcription analysis | GATGAAGTCGGGTCGTGTAC |
| 6 | KAR2_RE |  |  | TCTTAGCAGCATCACCCAACC |
| 7 | TDH3_FW | TDH3 | Transcription analysis | GGTGAGGTTTCTGCCAGC |
| 8 | TDH3_RE |  |  | GTGGACTCAATGACGTAGTC |
| 9 | PGK1_FW | PGK1 | Transcription analysis | AGAACGGTGGAACTGTCATCGT |
| 10 | PGK1_RE |  |  | AAAGAAGCTCCTCCTCCAGTGG |
| 11 | HAC1_FW | HAC1 | Transcription analysis | CATTACAGCAGGCTCCATC |
| 12 | HAC1_RE |  |  | GTCAACTGATATGTGCCAAC |
